# Covariance of Phytoplankton, Bacteria, and Zooplankton Communities Within *Microcystis* Blooms in San Francisco Estuary

**DOI:** 10.3389/fmicb.2021.632264

**Published:** 2021-06-07

**Authors:** Peggy W. Lehman, Tomofumi Kurobe, Khiet Huynh, Sarah Lesmeister, Swee J. Teh

**Affiliations:** ^1^Division of Environmental Services, California Department of Water Resources, West Sacramento, CA, United States; ^2^Department of Anatomy, Physiology and Cell Biology, School of Veterinary Medicine, University of California, Davis, Davis, CA, United States

**Keywords:** *Microcystis*, cyanobacteria blooms, phytoplankton, bacteria, metagenomics, estuary, zooplankton, gene sequencing

## Abstract

*Microcystis* blooms have occurred in upper San Francisco Estuary (USFE) since 1999, but their potential impacts on plankton communities have not been fully quantified. Five years of field data collected from stations across the freshwater reaches of the estuary were used to identify the plankton communities that covaried with *Microcystis* blooms, including non-photosynthetic bacteria, cyanobacteria, phytoplankton, zooplankton, and benthic genera using a suite of analyses, including microscopy, quantitative PCR (qPCR), and shotgun metagenomic analysis. Coherence between the abundance of *Microcystis* and members of the plankton community was determined by hierarchal cluster analysis (CLUSTER) and type 3 similarity profile analysis (SIMPROF), as well as correlation analysis. *Microcystis* abundance varied with many cyanobacteria and phytoplankton genera and was most closely correlated with the non-toxic cyanobacterium *Merismopoedia*, the green algae *Monoraphidium* and *Chlamydomonas*, and the potentially toxic cyanobacteria *Pseudoanabaena*, *Dolichospermum*, *Planktothrix*, *Sphaerospermopsis*, and *Aphanizomenon*. Among non-photosynthetic bacteria, the xenobiotic bacterium *Phenylobacterium* was the most closely correlated with *Microcystis* abundance. The coherence of DNA sequences for phyla across trophic levels in the plankton community also demonstrated the decrease in large zooplankton and increase in small zooplankton during blooms. The breadth of correlations between *Microcystis* and plankton across trophic levels suggests *Microcystis* influences ecosystem production through bottom-up control during blooms. Importantly, the abundance of *Microcystis* and other members of the plankton community varied with wet and dry conditions, indicating climate was a significant driver of trophic structure during blooms.

## Introduction

As a powerful competitor in the water column, and the most common harmful cyanobacteria bloom species in freshwater, the presence of the cyanobacterium *Microcystis* spp. shapes freshwater plankton communities and ecosystem processes worldwide (Harke et al., 2016; Paerl et al., 2018). The ability of *Microcystis* to compete effectively for key resources is well known. A high ammonium and inorganic carbon uptake rate, the ability to use urea as a nitrogen source, cellular storage of phosphate, sequestering of inorganic and organic compounds, and daily vertical migration through buoyancy regulation to access nutrients throughout the water column makes *Microcystis* a strong competitor for nutrients (Belisle et al., 2016; Harke et al., 2016; Paerl and Otten, 2016). *Microcystis* also competes through resource partitioning by migrating to the surface where it can grow at both high light and water temperature (WT) that many species cannot (Paerl and Huisman, 2009; Huisman et al., 2018).

Less well studied is the species-specific interaction of *Microcystis* with other members of the plankton community, which may be critical to bloom development (Cook et al., 2020). *Microcystis* engages in resource exploitation and interference strategies to shape plankton communities (Dunker et al., 2013; Zhang et al., 2013; Bittencourt-Oliveira et al., 2015). Both the microcystin toxin and other secondary metabolites it produces, including lipopolysaccharides, inhibit phytoplankton, cyanobacteria, and non-photosynthetic bacteria through impacts on growth and cell membrane structure. These interactions are sufficiently sophisticated that they can lead to dose-response interactions between cells (Bittencourt-Oliveira et al., 2015; Song et al., 2017). Toxins and secondary metabolites in *Microcystis* are also powerful enough to affect the growth of higher trophic level organisms, including invertebrate predators (e.g., zooplankton) and fish, as well as higher aquatic plants (Jiang et al., 2011; Harke et al., 2016; Shumway et al., 2018). However, in order to survive, *Microcystis* must develop synergistic relationships (Cook et al., 2020). *Microcystis*, unlike many cyanobacteria, cannot fix nitrogen, and therefore, often relies on nitrogen fixing cyanobacteria in nutrient limited waters and ammonium from regeneration to provide the nitrogen needed for growth (Paerl and Otten, 2016). In addition, *Microcystis* has reduced metabolic functions and requires assistance to synthesize vitamins, degrade carbon, produce toxic metabolites, cycle nitrogen, and run transport systems (Xie et al., 2016; Cook et al., 2020). The versatility *Microcystis* displays to survive and shape the plankton community in its favor may partially explain why it is the most common of the freshwater cyanobacteria worldwide (Harke et al., 2016) and expected to increase with the adverse conditions associated with climate change (Paerl and Huisman, 2009).

*Microcystis* blooms first occurred in upper freshwater and brackish water reaches of upper San Francisco Estuary (USFE) in 1999 (Lehman et al., 2005). Its expansion over time suggests it adapts well to environmental conditions and competes well for limiting resources in the estuary. Although nutrients are commonly in excess in USFE, *Microcystis* responds rapidly to the periodic availability of ammonium, the preferred nitrogen source (Lee et al., 2015; Lehman et al., 2015, 2017). Very large diameter colonies (50 mm) allow *Microcystis* to float on the surface of the water column where light is plentiful in this turbid estuary (Lehman et al., 2013), and where they can rapidly disperse on the surface film with streamflow, wind, and tide across hundreds of kilometers of waterways. *Microcystis* tolerates the elevated water temperatures in the estuary that reach ≥25°C in the summer (Lehman et al., 2013, 2017) and varies most closely with water temperature and residence time (Lehman et al., in press). *Microcystis* blooms are commonly toxic and often contain the potentially most toxic microcystin, microcystin-LR (Lehman et al., 2008; Baxa et al., 2010). Microcystins or other toxic substances in the blooms may contribute to the low abundance of diatom and green algae (Lehman et al., 2010, 2017). These toxic substances and cell lysates may also alter the diversity and abundance of bacteria during blooms (Otten et al., 2017; Kurobe et al., 2018a). Microcystins occur within organisms throughout trophic levels in the food web (Lehman et al., 2005) and were correlated with a decrease in the survival of zooplankton (Ger et al., 2009, 2010) and native fish (Acuna et al., 2012, 2020; Kurobe et al., 2018b). An analysis of the planktonic communities within *Microcystis* blooms across trophic levels and how these change with wet and dry conditions has not been done.

The purpose of this study was to address the hypotheses that (1) phytoplankton, cyanobacteria, and non-photosynthetic bacteria community composition, as well as lower trophic level plankton (e.g., zooplankton) form distinct communities during *Microcystis* blooms and (2) that the abundance and toxicity of *Microcystis* and the co-occurring planktonic community vary with wet and dry conditions. Such information is needed to better understand, model, and manage controlling factors within *Microcystis* blooms that are expected to increase with the frequency and intensity of drought due to climate change (Dettinger et al., 2016).

To address these hypotheses, phytoplankton, cyanobacteria, non-photosynthetic bacteria (bacteria), zooplankton, and benthic communities were measured in field samples from 10 stations across the estuary using a suite of analyses, including microscopy, quantitative PCR (qPCR), and shotgun metagenomic analysis during *Microcystis* blooms between 2014 and 2018. These years were unique in that they included a wide range of environmental conditions. The water years 2014 and 2017 were the 3rd driest and the wettest water years on record since 1906 (Lehman et al., 2018, in press). The coherence of plankton within the *Microcystis* bloom season was quantified using agglomerative hierarchical cluster analysis (CLUSTER) with similarity profile analysis (SIMPROF) and correlation analysis. The potential influence of environmental conditions was determined using permutational multivariate ANOVA (PERMANOVA).

## Materials and Methods

### Site Description

**Figure 1:**
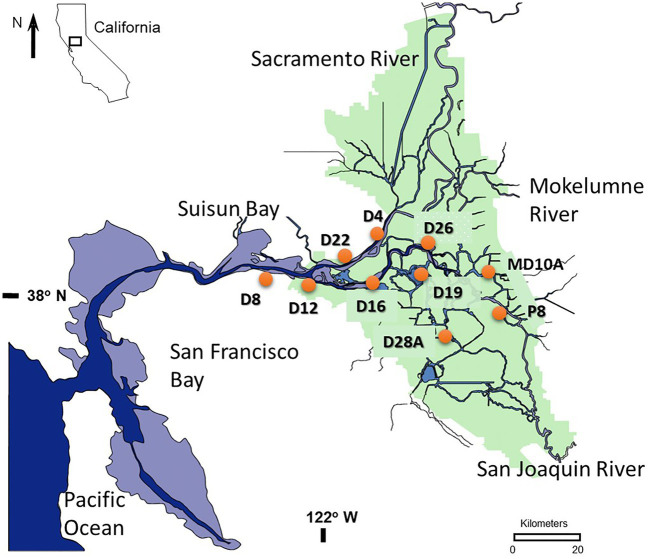
Map showing the location of 10 stations sampled between 2014 and 2018 in the upper San Francisco Estuary (USFE).

The USFE is comprised of a 2,990 km^2^ inland delta (Delta) containing 1,100 km of waterways and a shallow brackish water embayment called Suisun Bay (SB). The Delta is bounded by the Sacramento River on the north and the San Joaquin River on the south (Figure 1) and extends upstream to the head of tide at Freeport on the Sacramento River and Vernalis on the San Joaquin River. These two major rivers converge near Antioch and then flow to the Pacific Ocean through a chain of three marine bays – Suisun, San Pablo, and San Francisco. The Delta and three marine bays form San Francisco Estuary, the largest estuary on the west coast of North America. Water depth in the Delta varies from a few meters in shallow flooded islands and marine bays to 13 m in the center of major river channels. Tides reach 2 m in height; have velocities up to 30 cm s^−1^ and range 10 km or more during tidal excursion. Due to its Mediterranean climate summers are dry and the water year is based on precipitation from October to the following September (Swain et al., 2018). *Microcystis* blooms consistently occur during the dry summer and fall months between July and October (Lehman et al., 2017).

### Field Sampling

Data for this study were selected from multiple independent, but similar studies. Water samples for 2014 and 2015 were collected by van Dorn bottle for 10 stations at 2-week intervals and 0.3 m depth (Figure 1). Water samples for 2016 through 2018 were collected by a through-hull pump for 10 stations at 4-week intervals and 1 m depth. Across the years, a total of 309 samples were collected. Among these data, five of the stations (stations D19, D26, D28A, D4, and P8) were sampled consistently throughout both studies and totaled 116 samples.

Water temperature, pH, specific conductance, turbidity (NTU), and dissolved oxygen concentration were measured for each station within the first 0.3–1 m depth using a Yellow Springs Instrument (YSI) 6600 or EXO water quality sonde.

To fully quantify the *Microcystis* population, sampling was conducted for both the surface film which contained visible, widely dispersed, and wide diameter (>25 mm) colonies and the water just below the surface film with single cells and non-visible colonies. *Microcystis* colonies floating on the surface film were sampled by gentle hand tows of a plankton net (75 μm mesh, 0.5 m diameter) fitted with an Oceanic flow meter for 30.5 m (Lehman et al., in press). Previous research indicated this was the only way to quantitatively sample these populations. Subsurface samples for determining the cell abundance of total and potentially toxic cyanobacteria by qPCR, as well as all phytoplankton and cyanobacteria by microscopy were collected with whole water samples taken just below the surface film at 0.3–1 m (see above) using a van Dorn or thru-hull pump system. Both surface and subsurface water samples for identification and enumeration by microscopy were placed in amber glass bottles, preserved with Lugol’s solution, and kept at room temperature and in the dark until analysis. Subsurface water samples (200–300 ml) for qPCR analysis were filtered through nitrocellulose membrane filters (pore size 0.45 μm, Thermo Fisher Scientific, Waltham, Massachusetts) within 24 h of sample collection in a laboratory and frozen at −80°C until analysis.

### Water Quality Analyses

Water samples for ammonium, nitrate plus nitrite, silica, and soluble reactive phosphorus (SRP) analyses were filtered through nucleopore filters (0.45 μm pore size) and frozen until analysis (United States Environmental Protection Agency, 1983; United States Geological Survey, 1985; American Public Health Association et al., 2012). Water for dissolved organic carbon analysis was filtered through a pre-combusted GF/F filter (pore size 0.7 μm) and kept at 4°C until analysis (American Public Health Association et al., 2012). Unfiltered water samples for total suspended solids, total organic carbon, and total phosphate analyses were kept at 4°C until analysis (American Public Health Association et al., 2012).

### Phytoplankton and Cyanobacteria Composition

The abundance of *Microcystis* cells within the large colonies on the surface film was by determined by microscopy using a FlowCAM digital imaging flow cytometer (Fluid Imaging Technologies, Sieracki et al., 1998). FlowCAM estimates of cell abundance for these large colonies had a lower standard deviation (SD) than for qPCR analysis. For ease of analysis, the samples were subdivided into two size fractions, <300 and >300 μm, and images were counted at magnifications of 10X and 4X, respectively. The biovolume of each colony was measured using area-based diameter (ABD). Biovolume was converted to cell abundance based on the average ABD volume of single cells.

The abundance of single cells and small colonies for total cyanobacteria, total *Microcystis* and potentially toxic *Microcystis*, *Dolichospermum*, and *Aphanizomenon* in subsurface whole water samples was enumerated using qPCR. The qPCR analysis enabled us to determine the abundance of single cells too small to identify and enumerate quantitatively by microscopy. DNA extraction was performed on frozen filters using a NucleoSpin Plant II Kit (Macherey-Nagel, Bethlehem, PA, United States) according to the manufacturer’s instructions. The qPCR assays specific for *Dolichospermum*, *Aphanizomenon*, and total *Microcystis* (sum of toxic and non-toxic) targeted the 16S ribosomal RNA genes (16S rDNA). The abundance of toxic *Microcystis* was measured by quantifying the copy number of a microcystin synthetase gene (*mcy*D; Rinta-Kanto et al., 2005). Details of this analysis including primer and probe sequences are detailed in Lehman et al. (2017).

Natural units (individual cell, colony, or filament) of all phytoplankton and cyanobacteria in subsurface whole water samples were identified and enumerated using the Utermohl inverted microscope technique at 800X magnification (>2 μm cells; American Public Health Association et al., 2012). At least, 400 natural units were enumerated and identified to phyla and genera for each sample. These data were obtained from the California Department of Water Resources.^1^ Phytoplankton in this study refers to the photosynthetic phyla Bacillariophyta (diatoms), Chlorophyta (green algae), Chrysophyta (chrysophyte), Cryptophyta (cryptophyte), and Dinoflagellata (dinoflagellate). Photosynthetic bacteria in the phylum Cyanophyta are referred to as cyanobacteria.

### Toxin Analysis

Total microcystins concentration, a toxin commonly found in *Microcystis* and other potentially toxic cyanobacteria, was measured as microcystin-LR equivalents from the sum of the particulate (algal cells) and the dissolved fraction (water) in whole water samples collected at 0.3–1 m depth using a protein phosphatase inhibition assay (PPIA) kit (Product No. 520032, Eurofins-Abraxis, Warminser, PA, United States). Particulate and dissolved fractions were separated by filtering the whole water sample through a glass fiber membrane (934-AH, 1.5 μm particle retention size, Whatman, Little Chalfont, Buckinghamshire, United Kingdom). Particulate organic matter on the filter was subjected to microcystin extraction using 80% methanol, followed by dilution before quantification of microcystin by PPIA. The filtrate was used directly for PPIA analysis. Saxitoxin and anatoxin concentrations were measured by enzyme linked immunosorbent assay kits (Eurofins-Abraxis). The absorbance was measured using an Infinite M200 plate reader (TECAN, Mannedorf, Switzerland).

### Shotgun Metagenomic Analysis

Shotgun metagenomic analysis was conducted for a subset of the surface water samples to quantify the abundance and composition of the plankton community that covaried with *Microcystis* in the San Joaquin River, where *Microcystis* was most abundant. A range of values across space, time, and hydrologic conditions were obtained by analyzing the particulate organic matter for samples at three stations representing lower (station D12), middle (station D26), and upper (station P8) reaches of the San Joaquin River, the bloom season (June or July, August and October) and the 3 years (2014, 2016, and 2017) with low (2014) to high (2017) streamflow. For these samples (*n* = 27), particulate organic matter collected within surface water tows was filtered onto glass fiber membrane filters (934-AH, Whatman). Genomic DNA was extracted from half of the filter using a NucleoSpin Plant II Kit (Macherey-Nagel) according to the manufacturer’s instructions. The extracted genomic DNA was then submitted to the DNA Technology Core Facility at UC Davis for DNA sequencing reactions using a MiSeq instrument with paired-end 250 bp sequencing with Version 2 chemistry.^2^ The details for library preparation and sequencing reactions can be found in Kurobe et al. (2018a). The DNA sequencing data are available in the National Center for Biotechnology Information Database (www.ncbi.nlm.nih.gov/; BioProject ID: PRJNA434758; BioSample accession numbers: SAMN15573804–SAMN15573830).

A series of bioinformatics programs were used for the DNA sequence data processing. The sequence data in FASTQ format were subjected to quality check using FastQC ver. 0.11.5 (Andrews, 2010). Trimming and concatenating forward and reverse reads were done using PEAR ver. 0.9.10 (overlap length: 20 bp, minimum length: 250 bp; Zhang et al., 2014). After quality trimming and concatenating pair-end sequences, 76.6 million DNA sequences with an average length of 414 bp for the 27 libraries were obtained. The relative abundance of phyla and genera across trophic levels was determined using DNA sequences within BLASTN sequence similarity searches with a non-redundant nucleotide database downloaded from the NCBI website on August 16th, 2019 (cutoff values: bit score ≥200 and percent identity ≥95.00%).

Full taxonomic information for each organism was retrieved from the NCBI database using a R package, taxize ver. 0.9.0 and number of genus names was counted for each library using R ver. 4.0.1 (R Core Team, 2020). Rare taxa, defined as a percentage of DNA sequences less than 0.01% in the consolidated datasets, were not included in the data analysis. Phyla and genera annotated included non-photosynthetic prokaryotes in the domain Bacteria (bacteria), photosynthetic prokaryotes in the domain Bacteria and phylum Cyanophyta (cyanobacteria), phytoplankton (eukaryotes), Arthropoda and Rotifera (zooplankton), Mollusca (clams), and Porifera (sponges).

MEGAN ver. 6.20.6 (Community Edition) was used to identify the functions associated with DNA sequences within the clusters of orthologous groups (COG) of proteins database (eggNOG ver. 3.0) available in the software package (Powell et al., 2012; Huson et al., 2016). Prior to the functional analysis, similarity searches were performed by DIAMOND ver. 2.0.4 using the concatenated DNA sequences with a non-redundant protein database downloaded from the NCBI website on August 17th, 2020 (Buchfink et al., 2015). Normalized DNA sequence count data were used for (1) comparing COG categories among the 3 years; (2) assessing relative abundance of DNA sequences associated with cyanotoxin production and vertical migration in the 2014 data; and (3) investigating DNA sequences associated with nitrogen fixation and photosynthetic carbon reduction cycle (i.e., Calvin cycle) to identify sources of nitrogen (Supplementary Table T1). All the data processing for bioinformatics analysis was performed using a custom workstation built with 2x Xeon E5-2630 6 core CPU with 256GB ECC RAM, 4x HDD in RAID 10 configuration for Data Storage, and 64-bit Linux system with Ubuntu ver. 16.04 LTS.

### Statistical Analysis

Because phytoplankton data do not meet the assumptions of homogeneity of variance and normal distribution needed for parametric analysis, analyses were conducted with non-parametric statistics using the PRIMER-3 v. 7 software package (Clarke and Gorley, 2015). Single and multiple comparisons were computed using analysis of similarity (ANOSIM) of resemblance matrices for transformed (fourth root) biological data (zero adjusted Bray Curtis similarity coefficients) and normalized environmental data (Euclidean distance). Correlation was conducted with Spearman’s rank correlation (r). Clustering of abundance data was computed with CLUSTER using Whittaker’s Index of Association to identify clusters of genera or phyla that varied together among samples (r-mode; Somerfield and Clarke, 2013). Type 3 SIMPROF analysis identified genera or phyla within the clusters that were significantly different (5% significance level) and varied in a coherent manner across samples. Cluster analysis was conducted on abundance values which were standardized to the total abundance for each genus or phyla across all samples on a reduced set of species or phyla (>5% of the total abundance). Standardized values were also used to construct coherence curves for genera or groups of genera. Groups of genera or phyla were identified from dendrograms showing group-average clusters. Significant correlation between standardized genera and phyla abundance (Bray-Curtis similarity matrix) and environmental variables was determined using permutational multivariate ANOVA (PERMANOVA) distance-based linear modeling (DISTLM).

Comparison of the metagenomic analysis data (i.e., DNA sequence) assigned to each COG category among different years were performed using the Kruskal-Wallis Rank Sum Test, followed by pairwise comparison using the Wilcoxon Rank Sum Exact Test in R (R Core Team, 2020). Data average and SD units were also developed using R.

Modeled and measured streamflow variables (e.g., river flow, water diversion, and the X2 index of salinity intrusion) were obtained from the DAYFLOW database.^3^ The X2 index measures the distance (km) upstream from the Pacific Ocean where the bottom salinity is 2 and is used here as a proxy for residence time. Streamflow data and other environmental data used in multivariate analysis were reduced to those variables that were not highly correlated (*r* < 0.80). In addition, for ease of interpretation, stations were grouped into the following six regions based on station location: Suisun Bay (station D8), lower Sacramento River (LSAC; stations D4 and D22), lower San Joaquin River (LSJR; stations D12 and D16), central Delta (CD; stations D19 and D28A), east Delta (ED; station MD10A), and the south Delta (SD; stations D26 and P8).

## Results

### Temporal Patterns

Over the 5-year study, 2014 and 2015 were drought years with low average streamflow in the Sacramento and San Joaquin Rivers and high residence time (X2 index) compared with 2016 through 2018 (Table 1). The high streamflow in 2017 was higher than in 2014 for both the Sacramento (factor of 2) and San Joaquin (factor of 8) Rivers (*p* < 0.05). The high streamflow in 2017 was accompanied by significantly lower specific conductance and higher turbidity in 2017 compared with other years (*p* < 0.05). High turbidity caused by high suspended sediment concentration should have increased SRP and total phosphate concentration in 2017, because the sediments are rich in phosphorus. However, dilution caused by high streamflow produced the low SRP, total phosphate, total and dissolved organic carbon, dissolved organic nitrogen, and total dissolved solids in 2017 compared with 2014 (*p* < 0.05). The dry years 2014 and 2015 were characterized by relatively low ammonium concentration and high nitrate concentration compared with other years. Average water temperature was above 20°C for all years but increased after 2014.

**Table 1:** Average and SD of environmental variables measured at 10 stations between July and November for 2014–2018 in the USFE.

Variable	2014	2015	2016	2017	2018	Significant difference
Water temperature, °C	21.46 ± 1.2	20.24 ± 1.0	20.38 ± 1.02	20.66 ± 0.80	21.23 ± 1.30	2014 > 2016; 2016, 2018 > 2015; 2017 > 2016
Specific conductance, μScm^−1^	2,232 ± 3,182	2,288 ± 3,246	2,872 ± 4,198	1,138 ± 2076	2,586 ± 1794	2014, 2015, 2016, 2018 > 2017
Turbidity, NTU	5.23 ± 4.13	8.86 ± 11.17	9.14 ± 5.37	9.21 ± 6.01	8.14 ± 6.81	2016, 2017 > 2014, 2015; 2016, 2017 > 2018
pH unit	7.86 ± 0.20	7.78 ± 0.18	7.68 ± 0.14	7.54 ± 0.15	7.80 ± 0.18	2014, 2015, 2016, 2018 > 2017
Ammonium, mg L^−1^	0.04 ± 0.03	0.04 ± 0.03	0.06 ± 0.03	0.06 ± 0.02	0.06 ± 0.02	2016, 2017, 2018 > 2014; 2017 > 2015
Nitrate, mg L^−1^	0.55 ± 0.69	0.58 ± 0.62	0.32 ± 0.17	0.62 ± 0.94	0.34 ± 0.36	2014, 2015 > 2016, 2018; 2017 > 2014
Dissolved organic carbon, mg L^−1^	3.09 ± 0.82	3.06 ± 0.90	2.15 ± 0.69	2.20 ± 0.44	2.29 ± 0.75	2014, 2015 > 2016, 2017, 2018; 2017 > 2016; 2018 > 2017
Total organic carbon, mg L^−1^	3.22 ± 0.89	3.18 ± 0.86	2.14 ± 0.67	2.26 ± 0.45	2.32 ± 0.71	2014, 2015 > 2016, 2017, 2018; 2017 > 2016
Dissolved organic nitrogen, mg L^−1^	0.31 ± 0.10	0.30 ± 0.11	0.22 ± 0.05	0.16 ± 0.07	0.14 ± 0.08	2014, 2015, 2016 > 2017, 2018; 2015 > 2016
Soluble reactive phosphorus, mg L^−1^	0.15 ± 0.14	0.14 ± 0.13	0.08 ± 0.04	0.07 ± 0.03	0.10 ± 0.07	2014 > 2016, 2017, 2018; 2015, 2016 > 2017; 2018 > 2017
Total phosphorus, mg L^−1^	0.20 ± 0.22	0.20 ± 0.20	0.10 ± 0.04	0.08 ± 0.03	0.10 ± 0.07	2014, 2015 > 2016, 2017, 2018; 2016, 2018 > 2017
Silica, mg L^−1^	13.80 ± 1.08	11.98 ± 1.24	12.46 ± 1.07	13.14 ± 3.58	13.35 ± 1.44	2014 > 2015; 2017 > 2016
Total dissolved solids, mg L^−1^	1,472 ± 2,304	1,300 ± 1855	1,675 ± 2,516	640.95 ± 1,201	1,516 ± 2,292	2014, 2015, 2016, 2018 > 2017
Sacramento River streamflow, m^3^s^−1^	226 ± 30	204 ± 23	456 ± 83	471 ± 105	438 ± 89	All significant
San Joaquin River streamflow, m^3^s^−1^	14 ± 8	12 ± 10	18 ± 14	117 ± 67	28 ± 18	All significant
Water diversion, m^3^s^−1^	69 ± 25	53 ± 13	235 ± 40	262 ± 165	243 ± 61	All significant
X2 index, km	87 ± 2	86 ± 1	82 ± 2	75 ± 3	82 ± 1	All significant

Significant differences (*p* < 0.05) among years were calculated using analysis of similarity (ANOSIM) analysis. USFE, Significant differences.

**Figure 2:**
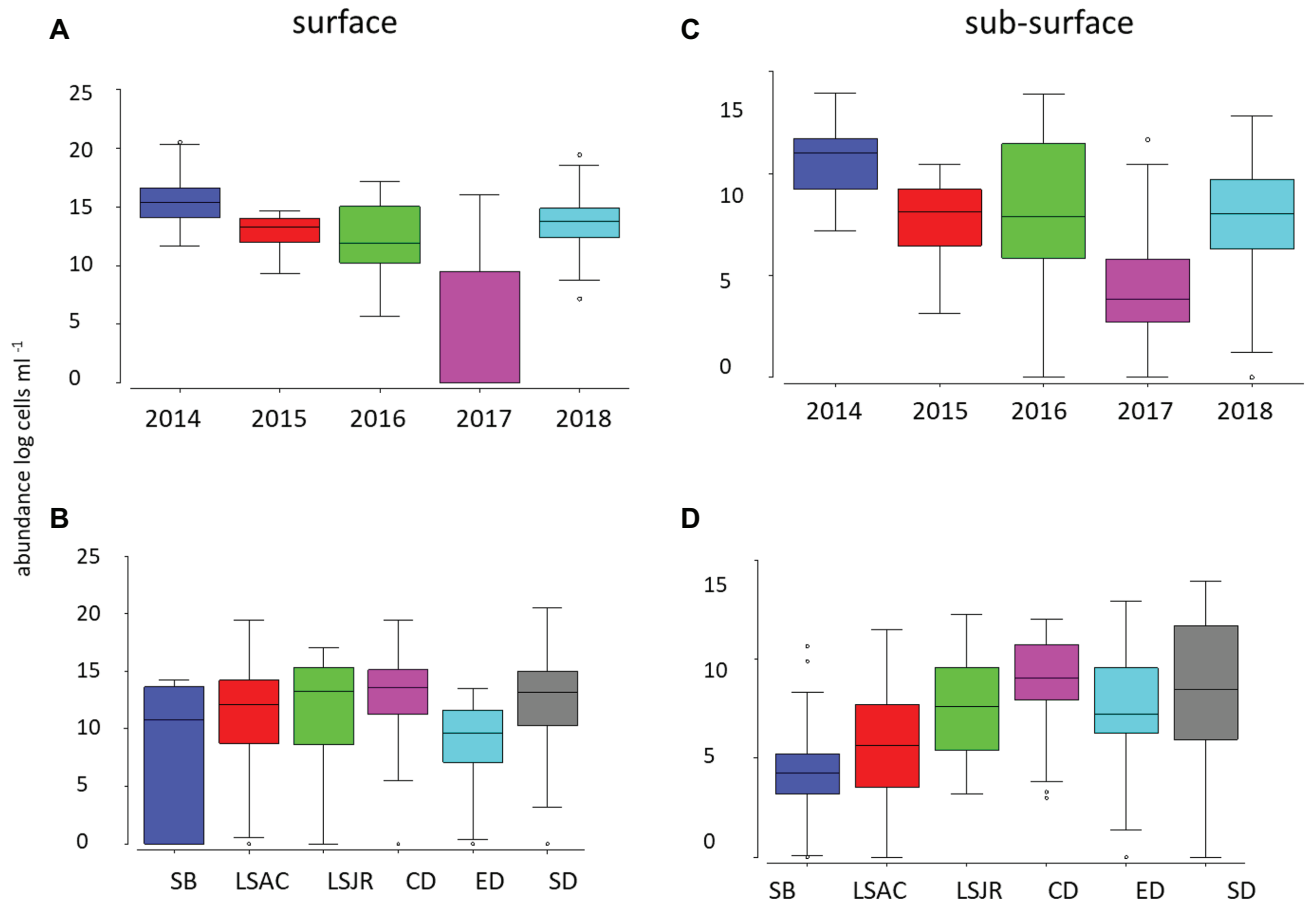
Average (line), 25th and 75th percentiles (box), and maximum and minimum (whiskers) concentration of *Microcystis* abundance for 2014 through 2018 at 10 stations in the USFE in surface net tows measured by microscopy among years **(A)** and among regions **(B)** and in the subsurface water by quantitative PCR (qPCR) among years **(C)** and regions **(D)**. Stations were grouped into the following six regions based on station location: Suisun Bay (SB, station D8), lower Sacramento River (LSAC, stations D4 and D22), lower San Joaquin River (LSJR, stations D12 and D16), central Delta (CD, stations D19 and D28A), east Delta (ED station MD10A), and the south Delta (SD, stations D26 and P8).

Surface *Microcystis* abundance was highest in 2014 and lowest in 2017 (*p* < 0.01; Figure 2A). There was no significant difference in *Microcystis* surface abundance among most of the remaining years, except for the higher abundance in 2018 than 2016 (*p* < 0.01). The total *Microcystis* abundance (single cells from qPCR) was lower in the subsurface water but demonstrated the same pattern as the surface; high and low abundance in 2014 and 2017, respectively (*p* < 0.05; Figure 2C).

Among regions, there was little difference in surface *Microcystis* abundance, except for the higher abundance in the central Delta and the south Delta than the east Delta (*p* < 0.05; Figure 2B). In contrast, subsurface *Microcystis* was greater in the south Delta than the east Delta, central Delta, LSAC, and Suisun Bay (*p* < 0.05; Figure 2D). The subsurface *Microcystis* abundance was also high in the central Delta compared with Suisun Bay, LSAC, LSJR, and the east Delta (*p* < 0.05).

**Figure 3:**
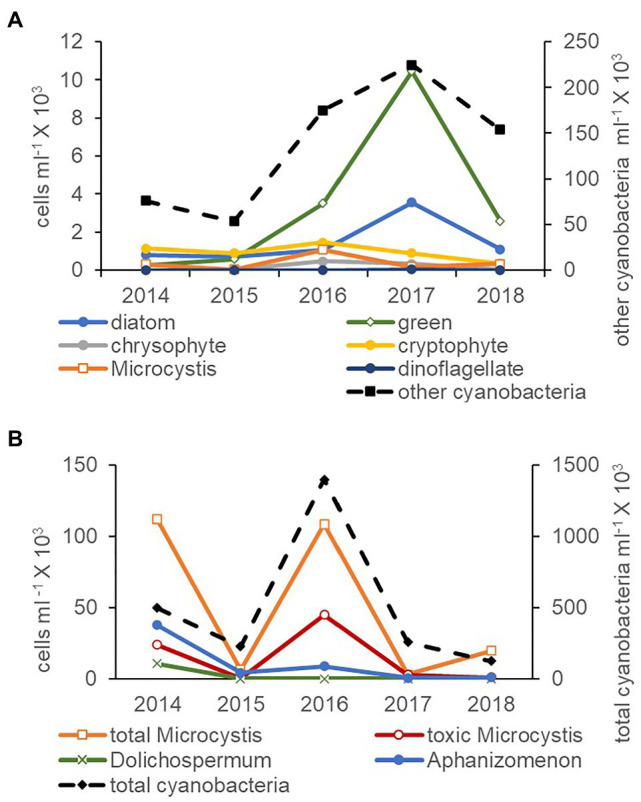
Average abundance of natural units for other cyanobacteria (non-*Microcystis*, secondary axis), *Microcystis* and phytoplankton measured by the inverted microscope technique **(A)** and total cyanobacteria (secondary axis), *Microcystis* and toxic cyanobacteria single cells measured by qPCR **(B)** for July through November between 2014 and 2018. Note the different axes scales.

Cyanobacteria were more abundant than all phytoplankton phyla combined (microscopy natural units) by at least an order of magnitude between 2014 and 2018 (Figure 3A). The high *Microcystis* abundance in 2016 was accompanied by an increase in the abundance of green algae. In the cyanobacteria community (qPCR single cells), total cyanobacteria were also more abundant than all three potentially toxic genera combined (Figure 3B). Total *Microcystis* abundance was greater in 2014 and 2016 and on average was comprised of 32% toxic *Microcystis* cells. An increase in the abundance of *Microcystis* cells in 2014 and 2016 was accompanied by an increase in the abundance of toxic *Microcystis*, total cyanobacteria, and Aphanizomenon cells (Figure 3B).

### Photosynthetic Community Associations

**Figure 4:**
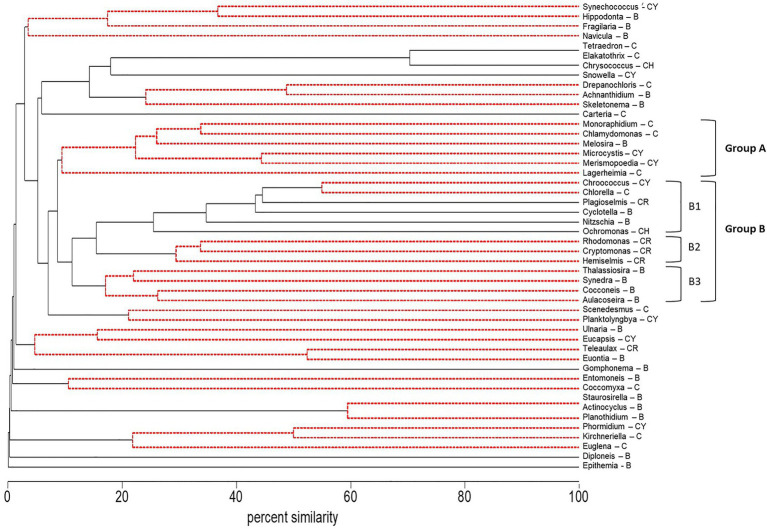
Dendrogram of group average clustering of abundance data for phytoplankton and cyanobacteria genera (microscopy) sampled between 2014 and 2018 at five stations. Differences among genera within clusters were determined by type 3 similarity profile analysis (SIMPROF) as significant (solid black line) or non-significant (dashed red line). Genera which were most coherent with *Microcystis* are coded as Group A. The next closest cluster of genera coherent with Group A was coded as Group B (with subsets B1, B2, and B3). Phyla associated with each genus are indicated by a letter code after the genus as follows: Bacillariophyta (diatoms, B), Chlorophyta (green algae, C), Chrysophyta (CH), Cryptophyta (CR), and Cyanobacteria (CY).

The abundance (microscopy) of the cyanobacteria, *Microcystis* and *Merismopoedia*, the diatom, *Melosira*, and the green algae, *Monoraphidium*, *Lagerheimia*, and *Chlamydomonas*, varied together based on cluster analysis and formed a coherent group of genera in Group A (Group A – *Microcystis*) that did not differ at the 5% level of significance from the 100% to the 10% similarity levels (dashed red lines, Figure 4). Correlation analysis supported the co-occurrence of *Microcystis* and *Monoraphidium* (*r* = 0.77; *p* < 0.01), *Merismopoedia* (*r* = 0.62; *p* < 0.01), *Chlamydomonas* (*r* = 0.51; *p* < 0.01), and *Melosira* (*r* = 0.29; *p* < 0.05) within Group A – *Microcystis*. In contrast, *Lagerheimia* was not significantly correlated with *Microcystis* or any of the other five genera in Group A – *Microcystis*.

**Figure 5:**
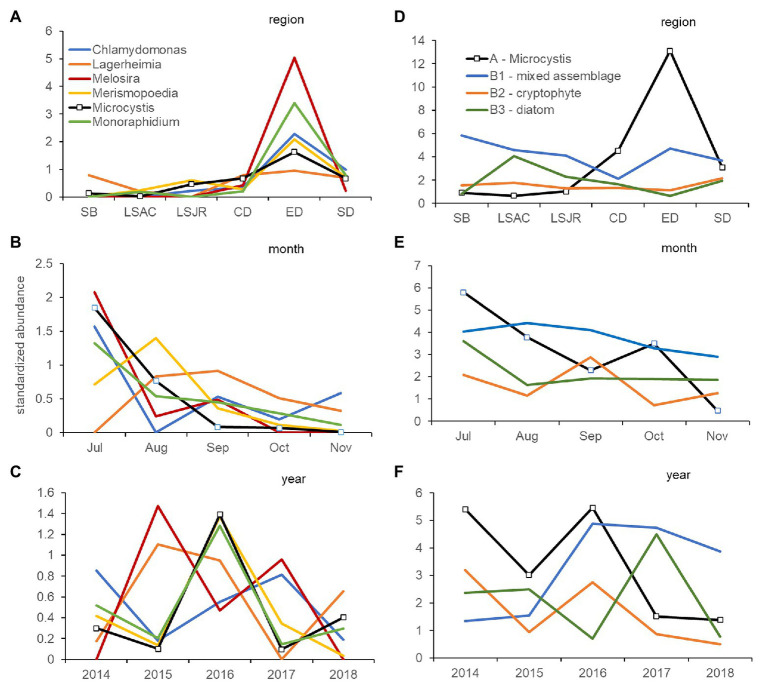
Coherence curves describing the average standardized abundance (microscopy) of phytoplankton and cyanobacteria genera from significantly different (5% level) groups of genera identified by SIMPROF within cluster analysis. Curves describe the variation of each genus in Group A, which includes *Microcystis*, by region **(A)**, month **(B)**, and year **(C)**. A second set of coherence curves describes the variation of the average standardized abundance for Group A, compared with Group B1 (mixed phyla), Group B2 (cryptophyte genera), and Group B3 (diatom genera) by region **(D)**, month **(E)**, and year **(F)**. Samples were collected monthly at 10 stations in the USFE between 2014 and 2018. Please note difference in scales among figures.

Coherence curves based on the average standardized abundance of genera, also indicated most genera within Group A – *Microcystis* varied together across regions (Figure 5A) and were abundant in mid-summer; July or August (Figure 5B). Among years, only *Microcystis*, *Merismopoedia*, and *Monoraphidium* consistently varied together and were greater in the dry years 2014, 2016, and 2018 than the wet year 2017 (Figure 5C). *Melosira* and *Chlamydomonas* were abundant during the 2017 wet year, while *Melosira* and *Lagerheimia* were abundant in the 2015 dry year.

The phytoplankton and cyanobacteria genera in Group A – *Microcystis*, were significantly different from the next closest cluster (percent similarity of 10%) of genera in Group B within the dendrogram (Figure 4). There were three subsets of genera in Group B. Group B1 (Group B1 – mixed) contained genera within multiple phyla: Badcillariophyta (*Nitzschia* and *Cyclotella*), Chrysophyta (*Ochromonas*), Cryptophyta (*Plagioselmis*), Chlorophyta (*Chorella*), and Cyanophyta (*Chroococcus*). Group B2 (Group B2 – cryptophyte) only contained Cryptophyta genera (*Hemiselmis*, *Cryptomonas*, and *Rhodomonas*), while Group B3 (Group B3 – diatom) only contained Bacillariophyta genera (*Aulacoseira*, *Cocconeis*, *Synedra*, and *Thalassiosira*).

Coherence curves based on the average standardized abundance of genera indicated genera in Group B1 – mixed were abundant in Suisun Bay and east Delta and contrasted with genera in Group A – *Microcystis* which were abundant in the central and south Delta regions (Figure 5D). Genera in Group B2 – cryptophytes were more abundant in September than those in Group A – *Microcystis*, which were abundant in July (Figure 5E). Among years, genera in Group A – *Microcystis* and Group B2 – cryptophyte were abundant during the 2014 and 2016 drought years (Figure 5F). Genera in Group B3 – diatom were abundant in 2017, while genera in Group B1 – mixed were abundant after 2015.

### Environmental Associations

The variation of genera within Group A – *Microcystis* was best explained by water temperature, specific conductance, ammonium, and total suspended solids based on DISTLM analysis (*n* = 185). Correlations between these variables and standardized abundance for Group A – *Microcystis* were positive and highest for water temperature (*r* = 0.38, *p* < 0.01) and negative and low for specific conductance (*r* = −0.22, *p* < 0.05), ammonium (*r* = −0.26, *p* < 0.01), and total suspended solids (*r* = −0.30, *p* < 0.01). Within Group A – *Microcystis*, water temperature was more closely correlated with *Microcystis* (*r* = 0.27, *p* < 0.01) than *Merismopoedia* (*r* = 0.25, *p* < 0.05), *Monoraphidium* (*r* = 0.18, *p* < 0.05), or *Melosira* (*r* = 0.18, *p* < 0.05).

In contrast, genera in Group B subgroups were primarily correlated with streamflow diversion variables. Significant explanatory variables from DISTLM analysis were: Group B1 – mixed (State Water Project diversion and Central Valley Project diversion); Group B2 – cryptophyte (dissolved organic nitrogen and Central Valley Project diversion); and Group B3 – diatom (Contra Costa Canal diversion, Central Valley Project diversion, specific conductance, and north bay aqueduct diversion). Because streamflow and agricultural diversion variables are closely correlated, these explanatory variables were the inverse of residence time. The State Water Project, Central Valley Project, north bay aqueduct, and Contra Costa Canal agricultural diversion variables were negatively correlated with the X2 index (*r* = −0.58, *r* = −0.61, *r* = −0.38 and *r* = −0.21, respectively; *p* < 0.01, *n* = 185).

### Toxin Concentration

**Figure 6:**
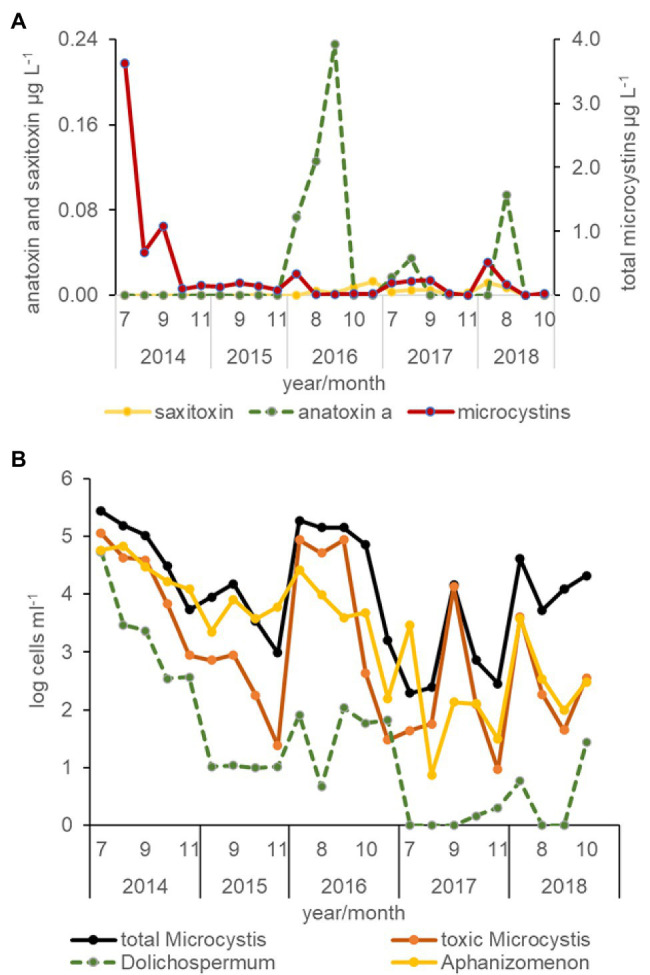
Average concentration of cyanotoxins determined by protein phosphate inhibition assay (PPIA) analysis **(A)** and the abundance of total *Microcystis*, toxic *Microcystis*, *Dolichospermum*, and *Aphanizomenon* cells for all stations by month and year between 2014 and 2018 determined from qPCR analysis **(B)**.

All three cyanotoxins, microcystin, anatoxin *a*, and saxitoxin were measured in the USFE beginning in 2016. Among these toxins, total microcystins concentration was measured more frequently above the limit of detection than anatoxin *a* or saxitoxin (Figure 6A). The highest total microcystins concentration occurred during July and was greater in the 2014 drought year than other years (*p* < 0.01). In contrast, relatively high concentrations of anatoxin *a* were measured in 2016 and 2018 during August and September. Low saxitoxin concentration characterized the USFE, which reached above the limit of detection in 2016. Saxitoxin concentration was measured more frequently in south Delta and the LSAC (not shown).

Total microcystins concentration varied directly with total *Microcystis* abundance for all data collected (*r* = 0.99, *p* < 0.01, *n* = 348) but was not strongly correlated with the abundance of toxic *Microcystis* cells identified by qPCR (*r* = 0.34, *p* < 0.01). Total microcystins concentration was also strongly correlated with the abundance of *Dolichospermum* (*r* = 0.53, *p* < 0.01), but was poorly correlated with the abundance of *Aphanizomenon* (*r* = 0.17, *p* < 0.01; Figure 6B). The abundance of toxic *Microcystis* was strongly correlated with *Dolichospermum* cells (*r* = 0.74, *p* < 0.01) and somewhat with its toxin, anatoxin *a* (*r* = 0.33, *p* < 0.01). Saxitoxin concentration, a common toxin in *Aphanizomenon*, was weakly correlated with total microcystin concentration (*r* = 0.26, *p* < 0.01) in the south Delta and LSAC beginning in 2016.

Based on DISTLM analysis, total microcystins concentration was best, but poorly explained by water temperature (WT, *r* = 0.22, *p* < 0.05) and residence time based on the negative correlation with the variable that described water diversion at the Central Valley Project (*r* = −0.20, *p* < 0.01). In contrast, anatoxin *a* concentration was greater when residence time was short or when water diversion increased for the Contra Costa Canal (*r* = 0.37, *p* < 0.01). The importance of streamflow and water temperature was suggested by the strong and positive correlation between Contra Costa Canal water diversion and a suite of DAYFLOW water diversion variables, including the State Water Project, Georgiana Cross Channel flow, and Diversion, as well as water temperature (*r* = 0.60, *r* = 0.73, *r* = 0.69, *r* = 0.44, *p* < 0.01, respectively). Saxitoxin concentration was explained by an increase in specific conductance (*r* = 0.19, *p* < 0.01), total suspended solids (*r* = 0.17, *p* < 0.01), and streamflow from the Cosumnes River in the east Delta (*r* = 0.23, *p* < 0.01), but low correlations emphasized the high variability of these data. The Cosumnes River flow variable was correlated with streamflow from the Mokelumne River (*r* = 0.46, *p* < 0.01) and east Delta (*r* = 0.35, *p* < 0.01), suggesting the importance of the east Delta streamflow to saxitoxin development.

### Metagenomic Analysis

**Figure 7:**
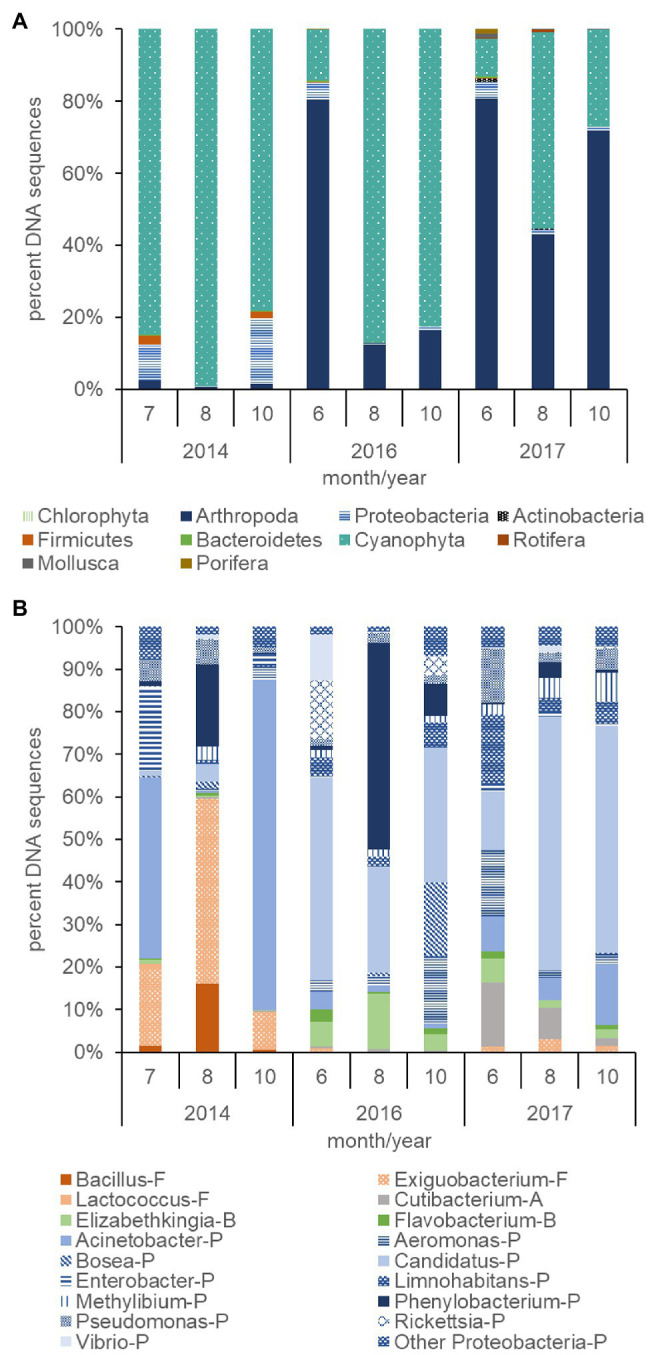
Percent DNA sequences of phyla for non-photosynthetic bacteria (Proteobacteria, Firmicutes, Bacteroidetes, and Actinobacteria), phytoplankton (Chlorophyta), cyanobacteria (Cyanophyta), zooplankton (Arthropoda and Rotifera), mollusks (Mollusca), and sponges (Porifera; **A**), and the percent of DNA sequences of non-photosynthetic bacteria genera **(B)** computed from surface samples collected during the summer and fall of 2014, 2016, and 2017. Letter codes and colors indicate the phyla for each bacterium: Proteobacteria (P, blue), Firmicutes (F, orange), Actinobacteria (A, gray), and Bacteroidetes (B, green). The other Proteobacteria category contains Proteobacteria that summed less than 5% of the total count each day.

The plankton community across trophic levels varied with Cyanophyta abundance during 2014, 2016, and 2017. The percent of Cyanophyta DNA sequences (78–99%) within the plankton community were relatively large during the summer and fall months of the 2014 dry year and contrasted with the relatively low percent of Cyanophyta DNA sequences (10–54%) in the summer and fall months of the 2017 wet year (*p* ≤ 0.01; Figure 7A). A greater percentage of Proteobacteria DNA sequences also occurred in the plankton in 2014 (9%) than 2017 (2%, *p* ≤ 0.05). Firmicutes DNA sequences were low overall but were greater (2%) in 2014 than 2017 (<1%, *p* ≤ 0.05). The composition of the plankton community during 2016, an intermediate streamflow year compared with 2014 and 2017, had an intermediate number of DNA sequences for Cyanophyta (14–87%) and did not differ significantly from 2014 to 2017 for Cyanophyta, Proteobacteria, or Firmicutes. The percent DNA sequences for Arthropoda were over 20 times lower during 2014 (2%) compared with 2016 (36%) and 2017 (65%, *p* ≤ 0.01, Figure 7A). In contrast, the average percent of DNA sequences for Rotifers, Porifera, Mollusca, and Bacteriodetes were low (<1%), variable and not significantly different among years.

The total number of DNA sequences per sample for bacteria in the 2014 dry year (253,619) exceeded those in 2016 (6,459) and 2017 (4,965) by orders of magnitude (not shown, *p* ≤ 0.05). In addition, the relatively large Cyanophyta blooms in 2014 and 2016 (Figure 7A) had a greater average percent of the xenobiotic bacterium *Phenylobacterium* (7–19%, Figure 7B) compared with 2017 (2%, *p* ≤ 0.05). Exiguobacterium was also elevated in 2014 (24%) compared with 2017 (2%, *p* ≤ 0.01). The Cyanophyta blooms in 2016 and 2017 differed from those in 2014 due to the presence of a high percentage (39–42%) of *Candidatus* (Proteobacteria/Actinobacteria) compared with 2014 (2%, *p* ≤ 0.01). In addition, the bacteria differed among months. The percentage of *Phenylobacterium* peaked in August for both years (19% for 2014 and 48% for 2016). In addition, the peak of the bloom in August 2014 and 2017 was preceded and followed by an elevated percentage of *Acinetobacter*, but this was not significant.

**Figure 8:**
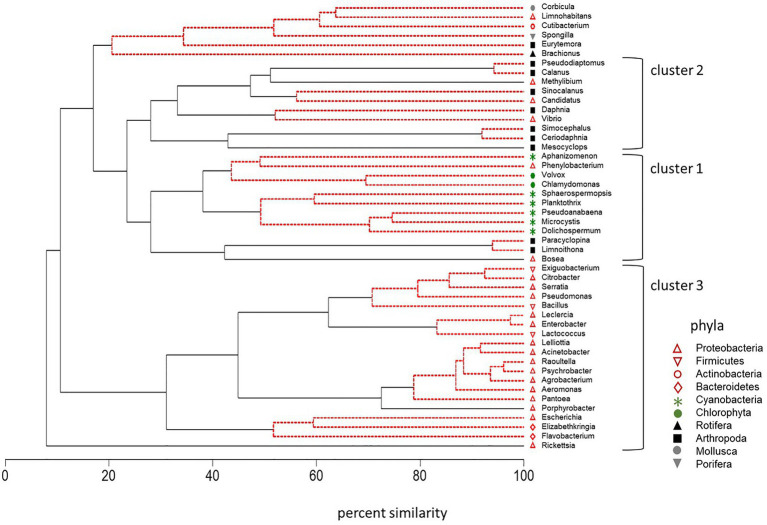
Dendrogram of cluster analysis combined with SIMPROF of DNA sequences for 10 phyla across trophic levels in surface samples collected in the summer and fall of 2014, 2016, and 2017. Type 3 SIMPROF (5% significance level) identified phyla that were significantly different (black solid line) or not significantly different (red dashed line).

CLUSTER analysis along with SIMPROF analysis of standardized counts for DNA sequences was also used to identify groups of plankton that behaved in a coherent manner across multiple trophic levels. *Microcystis* abundance varied with other cyanobacteria and green algae (Cluster 1, Figure 8). High correlation between *Microcystis* and the cyanobacteria *Dolichospermum* (*r* = 0.72, *p* < 0.01), *Pseudoanabaena* (*r* = 0.79, *p* < 0.01), *Planktothrix* (*r* = 0.72, *p* < 0.01), *Sphaerospermopsis* (*r* = 0.68, *p* < 0.01), and *Aphanizomenon* (*r* = 0.43, *p* < 0.05) supported the strong coherence of these genera within the cluster. *Microcystis* was also positively correlated with the green alga *Chlamydomonas* (*r* = 0.58, *p* ≤ 0.01) in Cluster 1. The Proteobacteria *Phenylobacterium* and *Bosea* were the only bacteria that occurred in Cluster 1. The SIMPROF analysis indicated the variation in the abundance of the *Phenylobacterium* bacterium was indistinguishable from that of the cyanobacteria and green algae *Aphanizomenon*, *Chlamydomonas*, and *Volvox* in the dendrogram. Correlation analysis also confirmed a positive correlation between the abundance of the bacterium *Phenylobacterium* and the cyanobacteria and green algae in Cluster 1: *Aphanizomenon*, *Chlamydomonas*, and *Microcystis* (*r* = 0.43 to *r* = 0.74, *p* ≤ 0.05). The bacterium *Bosea* was significantly correlated with *Dolichospermum*, *Microcystis*, and *Planktothrix* in Cluster 1 (*r* = 0.39 to *r* = 0.43, *p* ≤ 0.05). It was also closely correlated (percent similarity of 40%) with the small cyclopoid copepods *Limnoithona* and *Paracyclopina* on the dendrogram (*r* = 0.48 and *r* = 0.47, *p* ≤ 0.05). These small zooplankton were also positively correlated with the abundance of *Microcystis* (*r* = 0.46 to *r* = 0.51, *p* ≤ 0.01).

Cluster 2 contained the large calanoid copepods, *Pseudodiaptomus*, *Calanus*, and *Sinocalanus* (Arthropoda; Figure 8). Opposite to the small copepods, *Microcystis* abundance was negatively, correlated with the abundance of the large calanoid copepods *Calanus* (*r* = −0.48, *p* ≤ 0.05) and *Pseudodiaptomus* (*r* = −0.44, *p* ≤ 0.05). The correlation between the abundance of *Microcystis* and *Corbicula*, a large molluska grazer, was also negative (*r* = −0.51, *p* < 0.01).

Cluster 3 contained a suite of bacteria in the phyla Proteobacteria, Bacteriodetes, and Firmicutes (Figure 8). Among the Bacteriodetes, *Elizabethkingia* was negatively correlated with *Aphanizomenon*, *Dolichospermum*, and *Pseudoanabaena* (*r* = −0.38 to *r* = −0.45, *p* ≤ 0.05) while *Flavobacterium* was positively correlated with *Aphanizomenon* and *Dolichospermum* (*r* = 0.44 to *r* = 0.46, *p* ≤ 0.01). The Firmicutes bacteria were not correlated with the cyanobacteria in Cluster 1. Among the other Proteobacteria, *Agrobacterium* was correlated with *Planktothrix* (*r* = 0.51, *p* ≤ 0.01).

**Figure 9:**
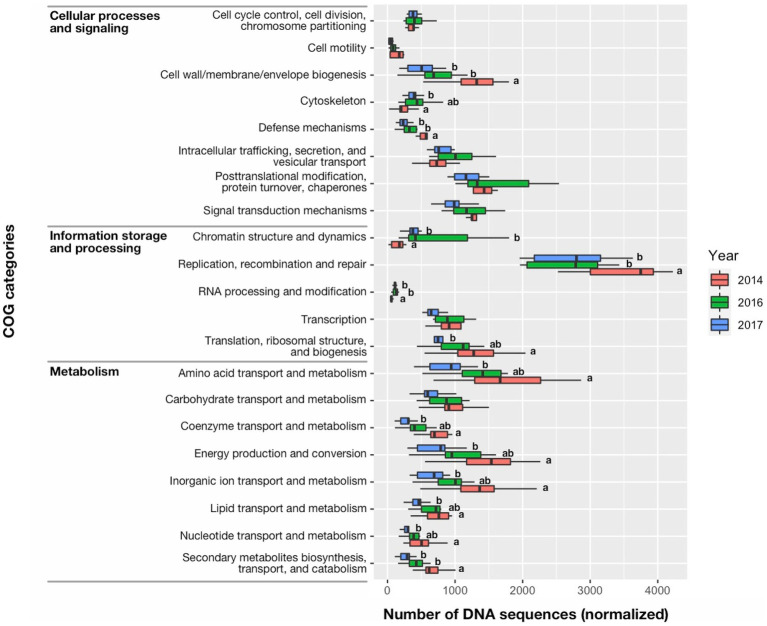
Median (bar), 25th and 75th confidence intervals (box), and maximum and minimum (whiskers) of DNA sequence counts in the clusters of orthologous groups (COG) of proteins database for cell functions of prokaryotes and eukaryotes. Significant differences among years were identified with a Kruskal-Wallis Rank Sum Test, followed by a pairwise comparison using a Wilcoxon Rank Sum Exact Test. Significant differences between years (<0.05 level of significance) were indicated by different letter codes.

The greater percent abundance (≥three times) of the Cyanophyta during 2014 compared with 2016 and 2017 was also characterized by differences in the quantity of DNA sequences among functional groups (Figure 9). DNA sequences associated with Information storage and processing: “Replication, recombination, and repair” and “Translation, ribosomal structure, and biogenesis” accounted for most of the metagenomic DNA in 2014 and was greater in 2014 than other years (*p* < 0.05). Similarly, the 2014 bloom was characterized by many DNA sequences associated with Metabolism: “Secondary metabolites biosynthesis, transport, and catabolism,” “Nucleotide transport and metabolism,” “Lipid transport and metabolism,” “Inorganic ion transport and metabolism,” “Energy production and conversion,” “Coenzyme transport and metabolism,” and “Amino acid transport and metabolism that were greater in 2014 than 2017 (*p* < 0.05).” Among the DNA sequences associated with Cellular processes and signaling: “Cell wall/membrane envelope biogenesis” was also greater in 2014 than other years (*p* < 0.01), as might be expected for rapidly growing cells. Interestingly, the highly toxic cyanobacteria bloom in 2014 also had more DNA sequences associated with “Defense mechanisms” than other years (*p* < 0.01). The COG category, “Secondary metabolites biosynthesis, transport, and catabolism” in 2014 was dominated by genes encoding both polyketide synthase modules and related proteins (eggNOG ID: COG3321) (1) non-ribosomal peptide synthetase (COG1020), and (2) calcium-binding proteins (COG2931) that support both microcystin production and vertical migration (Supplementary Figure S1; Kaebernick and Neilan, 2001; Drugă et al., 2019). The number of DNA sequences for the *Microcystis* and *Aphanizomenon* dominated bloom in 2016 were not significantly different than 2014 for Information storage and processing: “Translation, ribosomal structure, and biogenesis,” Cellular processes and signaling: “Cell motility” and “Cytoskeleton” and a large suite of sequences associated with Metabolism: “Energy production and conversion,” “Amino acid transport and metabolism,” “Nucleotide transport and metabolism,” “Coenzyme transport and metabolism,” and “Lipid transport and metabolism,” and “Inorganic ion transport and metabolism.” The lowest total number of DNA sequences associated with Information storage and processing, Cellular processes and signaling and Metabolism (42,693, 42,815, and 37,216, respectively) occurred in 2017, which also had the lowest *Microcystis* abundance. Infrequent and few DNA sequences associated with nitrogen fixation occurred in the metagenome data for this study and contrasted with the constant and moderately high number of DNA sequences associated with photosynthetic carbon reduction (i.e., Calvin cycle; Supplementary Figure S2). This suggested photosynthetic plankton were potentially using dissolved nitrogen in the ambient water for growth rather than nitrogen fixed by diazotrophs.

## Discussion

### Plankton Communities

Coherent phytoplankton and cyanobacteria assemblages characterized *Microcystis* blooms in USFE. *Microcystis* can influence the growth of phytoplankton and other cyanobacteria within blooms by excretion of substances that inhibit or stimulate growth through allelopathy (Kearns and Hunter, 2001; Singh et al., 2001; De Figueiredo et al., 2006; Suikkanen et al., 2006; Dunker et al., 2013; Zhang et al., 2013; Bittencourt-Oliveira et al., 2015; Song et al., 2017; Wang et al., 2017). Allelopathy was suggested in this study by the positive association between *Microcystis* and cryptophyte abundance and the negative association between *Microcystis* and both diatom and green algae abundance. Similar associations between *Microcystis* and green algae and diatoms were also measured for *Microcystis* blooms in USFE in 2004, 2005, and 2014 (Lehman et al., 2008, 2010, in press). The negative influence of *Microcystis* on diatoms and green algae and positive correlation with cryptophytes were also measured for microplankton in Vela Lake, Portugal (De Figueiredo et al., 2006) and in laboratory studies (Wang et al., 2017).

The increase in the abundance of the cyanobacteria *Microcystis* and *Merismopedia* and the green alga *Monoraphidium* with water temperature, particularly in the Delta where water temperatures reach beyond the tolerance of most microplankton (>20°C, Huisman et al., 2018), suggests species-specific interactions among these genera might have been synergistic. A close association between *Merismopoedia* and *Microcystis* was also measured for USFE in 2005 (Lehman et al., 2010). *Merismopoedia* survives in freshwater by creating trophic and symbiotic relationships with other aquatic organisms (Bukharin et al., 2001). A potential symbiotic relationship might be expected between *Merismopedia* and *Microcystis* because cyanobacteria can create water quality conditions that both promote other cyanobacteria (Sedmak and Elersek, 2005; Suikkanen et al., 2006) and reduce competition (Paerl and Otten, 2013, 2016). Allelopathic interactions can be strong for green algae too (Zhang et al., 2013). *Microcystis* inhibits *Monoraphidium* growth, but the reverse occurs as well, allowing both to coexist in the same environment (Bittencourt-Oliveira et al., 2015). The interaction between green algae and *Microcystis* can be complex though. In Lake Taihu, China competition between *Microcystis* and the green alga *Chlorella* led to an escalating dose-response interaction that enabled both to survive (Song et al., 2017).

### Metagenomics

DNA sequence data demonstrated the potentially broader impact of *Microcystis* blooms on the trophic structure in plankton communities. *Microcystis* covaried closely with a suite of potentially toxic cyanobacteria that combined could have affected planktonic competitors (Ibelings and Havens, 2008; Paerl et al., 2018). The coherence of *Microcystis*, *Aphanizomenon*, and *Dolichospermum* abundance measured for this study was first measured during the 2014 drought in USFE (Lehman et al., 2017). These three species commonly occur together in succession to dominate blooms (Paerl and Otten, 2016). Nitrogen fixation by *Dolicospermum* or *Aphanizomenon* is often necessary before *Microcystis*, a non-nitrogen fixing genus, can develop. However, nitrogen fixation was probably not needed in USFE, where the low number of DNA sequences suggested the photosynthetic plankton were potentially using nitrogen dissolved in the water rather than nitrogen fixed by diazotrophs. Previous research also suggested total nitrogen in the USFE was in excess due to elevated concentrations, ready uptake of both ammonium and nitrate, and a negative correlation between nitrogen and cyanobacteria biomass (Lehman et al., 2015; Cloern et al., 2020). This excess nitrogen enabled all three genera to occur together. As a result, these cyanobacteria may have species-specific connections that facilitate coexistence beyond nutrient concentration. A cooperative association between species was also suggested by the covariance of *Pseudoanabaena* and *Microcystis* abundance in the surface layer. Both *Pseudoanabaena* and *Dolichospermum* were identified as covariates with *Microcystis* in metagenomic analysis conducted for the flooded Mildred Island in USFE in 2012 (Otten et al., 2017). *Pseudoanabaena* was similarly identified as a covariate with *Microcystis* in the Nakdong River, Korea and the Villerest reservoir, France (Parveen et al., 2013; Chun et al., 2019). Recent research suggests that the occurrence of specific *Microcystis* genotypes with *Pseudoanabaena* and other bacteria in the Daechung Reservoir, Korea form pre-bloom, bloom, and post-bloom assemblages that influence seasonal succession of the blooms (Chun et al., 2020).

The negative correlation between the abundance of the large calanoid copepods Sinocalanus, *Calanus*, and *Pseudodiaptomus* with *Microcystis* abundance suggests the cyanobacteria assemblage may adversely affect the abundance of secondary producers in the plankton community. *Microcystis* and other cyanobacteria can adversely affect zooplankton survival through production of cyanotoxins as well as a suite of metabolites (Ibelings and Havens, 2008). Both cyanotoxins and protease inhibitors are known to affect factors such as food avoidance, food digestion, and molting in zooplankton (Martin-Creuzburg and Elert, 2004; Rohrlack et al., 2004, 2005; Hansson et al., 2007; Shumway et al., 2018). For USFE, the impact of *Microcystis* on lower food web trophic structure was demonstrated with bioassay studies in which the mortality and the species composition of copepods varied with both toxic and nontoxic strains of *Microcystis* and dissolved and particulate microcystins (Ger et al., 2009, 2010). In addition, the high abundance of total cyanobacteria and lack of diatoms during the *Microcystis* blooms provide poor quality food for zooplankton due to the lack of omega-3 fatty acids which are essential for growth, but are low in cyanobacteria (Galloway and Winder, 2015). The absence of large non-motile phytoplankton (e.g., diatoms) may partially explain the positive association between *Microcystis* and the small cyclopoid copepods *Limnoithona* and *Paracylopina*, which feed on ciliates and motile species (e.g., heterotrophs; Bouley and Kimmerer, 2006). The impact of cyanobacteria and their toxins on the food web may extend beyond zooplankton to the benthic community where mollusks were also negatively correlated with *Microcystis* abundance. Previous research demonstrated the potential impact of *Microcystis* across trophic levels due to the presence of microcystins and their effects on invertebrates (e.g., copepods, amphipods, worms, and jellyfish), mollusks, and fish in USFE (Lehman et al., 2005, 2010).

That only a few Proteobacteria and Bacteroidetes bacteria varied closely with *Microcystis* suggests the bloom may influence bacterial growth and diversity. Low diversity and abundance of bacteria during *Microcystis* blooms is common (Parveen et al., 2013; Chun et al., 2019) and was previously measured for USFE (Otten et al., 2017). The few Proteobacteria and Bacteroidetes bacteria that covaried with *Microcystis* during blooms have the potential to enhance nutrient recycling or breakdown of organic substances (Berg et al., 2009; Cook et al., 2020). The increase in DNA sequences associated with information storage and processing, metabolism, and cellular processes and signaling with cyanobacteria abundance also suggests the transfer of material and production of energy among prokaryotic and eukaryotic cells increases within a bloom. Proteobacteria and Bacteroides were also the most abundant bacteria during *Microcystis* blooms in Lake Erie and Grand Lake, OH (Steffen et al., 2012). Other bacteria, such as the Proteobacteria *Acinetobacter*, which were abundant before and after the large *Microcystis* bloom in August 2014, have been linked to the breakdown of cyanotoxins and organic matter (Berg et al., 2009). The Bacteroidetes bacterium *Flavobacterium* covaried with the cyanobacteria blooms but was not strongly correlated with *Microcystis*, and instead, was positively correlated with the cyanobacteria *Dolichospermum* and *Planktothrix*. In contrast, a negative correlation between *Flavobacterium* and *Microcystis* was calculated for Mildred Island in 2012 (Otten et al., 2017). These differing results support the potential importance of species-specific and site-specific interactions among closely correlated species in the cyanobacteria bloom. Recent research suggests that the *Microcystis* phycosphere is so variable that it varies among individual colonies over the season (Smith et al., 2021).

The variation of *Aphanizomenon*, *Microcystis*, and the green alga *Chlamydomonas* with the Proteobacteria bacterium *Phenylobacterium* was noteworthy. *Phenylobacterium* was also found to be closely correlated with the production of toxic *Microcystis* strains in Lake Taihu (Zuo et al., 2020). *Phenylobacterium* can obtain its energy for growth from xenobiotic compounds including those associated with herbicides (Eberspächer and Lingens, 2006). Herbicides are applied regularly in USFE to control aquatic weeds and can enter rivers through urban and agricultural runoff (Ta et al., 2017). The potential influence of herbicides on cyanobacteria and green algae growth in USFE was supported by a recent bioassay study in which the mortality of *Microcystis* and *Chlamydomonas* was less compared with the diatom *Thalassiosira* when exposed to Fluridone, a common herbicide used in USFE (Lam et al., 2019). Other bacteria, such as *Enterobacter*, *Elizabethkingia*, and *Raoultella* which were abundant during the blooms are common in water bodies worldwide and covary with activities associated with human contact. In general, human pathogens increase during *Microcystis* blooms (Chun et al., 2019).

### Trophic Cascade

The influence of *Microcystis* on bacteria during blooms could have further influenced trophic level production through impacts on the microbial loop (Segovia et al., 2015). Bacterial abundance and diversity decrease during *Microcystis* blooms (Woodhouse et al., 2016) and vary closely with the phases of the bloom (Bagatini et al., 2014). Heterotrophic nanoflagellates rely on bacteria as a source of food and nutrients and the variation of bacterial abundance with *Microcystis* affect the strength of the microbial loop (Moustaka-Gouni et al., 2006; Chen et al., 2010). The importance of the microbial loop in USFE was suggested by the variation of the small copepods *Limnoithona* and *Paracylopina* with *Microcystis*. These copepods may survive during *Microcystis* blooms because they eat small motile prey such as mixotrophic or heterotrophic ciliates and not the large, non-motile cells which characterize most cyanobacteria species (Bouley and Kimmerer, 2006). They may also thrive in the bloom environment which has few large zooplankton predators, such as *Pseudodiaptomus* or *Sinocalanus*. Although top-down predators are usually considered the primary control of plankton in USFE (Brown et al., 2016; Lucas et al., 2016), the negative correlation between *Microcystis* and plankton within multiple trophic levels (e.g., bacteria, diatoms, copepods, and mollusks) suggests the food web may also be influenced by bottom-up processes during blooms as well.

### Toxins

The simultaneous presence of microcystins, anatoxin *a*, and saxitoxin in 2016 through 2018 indicates summer blooms in USFE have matured through a succession from a simple *Microcystis* bloom containing only microcystins (Lehman et al., 2005, 2017) to a complex cyanobacterial harmful bloom with multiple species and toxins. This suggests there may be a synergism among these toxic species that may be promoted by the presence of toxins. Cyanobacterial blooms commonly contain multiple species and toxins worldwide, especially in large estuaries like the Chesapeake Bay (Paerl et al., 2018). Because anatoxin *a* and saxitoxin were not detected during *Microcystis* blooms sampled between 2004 and 2015 (Lehman et al., 2005, 2017), the presence of these toxins signals a shift in the bloom trajectory. The three toxins currently in the bloom may be partially due to the simultaneous abundance of *Microcystis*, *Dolichospermum*, and *Aphanizomenon*, which can produce this suite of toxins (Paerl et al., 2018). Positive correlations between *Dolichospermum* and *Aphanizomenon* and anatoxin *a* and saxitoxin supported the contribution of these genera to the toxin composition. However, high spatial and temporal variability characterizes cyanobacteria toxins in USFE (Baxa et al., 2010) and as the cluster analyses suggest, other species coherent with *Microcystis* may also contribute to the presence of these toxins in the water column.

### Climate Change

The importance of warm and dry conditions to plankton community composition across trophic levels supports the importance of climate change to the development and ecosystem impact of cyanobacteria blooms in USFE. The importance of warm water temperature and high residence time during dry years to the development of *Microcystis* blooms was supported by modeling studies in which water temperature and a proxy for residence time (X2 index) explained 78% of the variation in *Microcystis* abundance (Lehman et al., in press). Increased water temperature was also beneficial to the development of potentially toxic cyanobacteria and green algae that varied with *Microcystis* here and in previous studies (Lehman et al., 2017). Cyanobacteria are generally more tolerant of elevated water temperature, which gives them a competitive advantage over other plankton during drought (Huisman et al., 2018). Research also demonstrated cryptophytes and bacteria were more abundant during dry years (Lehman, 2000; Lehman et al., 2010; Kurobe et al., 2018a). Further, subtle differences among species may be important. The separation between *Microcystis* and *Aphanizomenon* within the cluster dendrogram, despite the fact that both were co-occurring cyanobacteria during blooms, may partially reflect a lower optimum water temperature for *Aphanizomenon*, which often leads to its occurrence earlier in the summer (Carey et al., 2012; Paerl and Otten, 2016). The importance of water temperature to the plankton community extends beyond the phytoplankton and cyanobacteria to the zooplankton, where the cyclopoid copepod *Limnoithona* probably increased in recent drought years partly due to its tolerance of warm water temperature (Bouley and Kimmerer, 2006). Residence time during dry years would not only affect the accumulation of plankton due to their limited mobility, but also the accumulation of chemical pollutants and bacteria, like *Phenylobacterium* that they support. Other water quality conditions which differ between wet and dry years, such as turbidity, would affect the plankton community structure through impacts on growth (Cloern and Jassby, 2012), and the success of species, like *Microcystis*, that can adapt to these conditions through strategies such as vertical migration or floatation (Paerl and Otten, 2013). Given the increased frequency of climate and anthropogenic induced drought in California (Dettinger et al., 2016), we can expect that the frequency and intensity of *Microcystis* blooms and their impact on the plankton community structure will continue to deepen, promoting bottom-up impacts on species, that will influence future estuarine production and management.

## Data Availability Statement

The datasets presented in this study can be found in online repositories. The names of the repository/repositories and accession number(s) can be found at: https://www.ncbi.nlm.nih.gov/genbank/, SAMN 15573804–SAMN15573830.

## Author Contributions

PL wrote the paper, analyzed phytoplankton, cyanobacteria, bacteria, and environmental data, directed field data collection, assisted with data collection, and was a principal investigator on the grant. TK and was a principal investigator, wrote methods for metagenomics, analysis, edited main text, analyzed data, and provided oversight and analysis of qPCR and DNA laboratory analysis. KH provided oversight for qPCR, toxin, and FlowCAM analysis. SL provided oversight of field sampling and ST was a principal investigator who managed the contractual elements and supervised the laboratory analyses. All authors contributed to the article and approved the submitted version.

### Conflict of Interest

The authors declare that the research was conducted in the absence of any commercial or financial relationships that could be construed as a potential conflict of interest.

## Footnotes

^1^https://water.ca.gov/Programs/Environmental-Services/Interagency-Ecological-Program

^2^http://dnatech.genomecenter.ucdavis.edu/

^3^https://water.ca.gov/Programs/Environmental-Services/Compliance-Monitoring-And-Assessment/Dayflow-Data

## Supplementary Material

Supplementary Figure S1 Profile of clusters of orthologous groups (COGs) found in the 2014 metagenome data.

Supplementary Figure S2 The number of DNA sequences associated with nitrogen fixation and the photosynthetic carbon reduction cycle (i.e., Calvin cycle) in all metagenome data.

Supplementary Table T1 List of clusters of orthologous groups (COGs) identification numbers used for the analyses in this study.
