# Supplementary material for: Covariance of Phytoplankton, Bacteria, and Zooplankton Communities Within Microcystis Blooms in San Francisco Estuary
Source: Front Microbiol. 2021 Jun 7;12:632264. doi: 10.3389/fmicb.2021.632264 (PMC8215387; doi:10.3389/fmicb.2021.632264)
Supplement: Supplementary Figure S1 — Profile of clusters of orthologous groups (COGs) found in the 2014 metagenome data. [file Presentation_1.pdf]

Supplementary Table T1. List of clusters of orthologous groups (COGs) identification numbers used for the analyses in this study.

Function	COG ID	Symbol	Description
Nitrogen fixation	COG2710*	NifD/CfbD	Nitrogenase Mo-Fe protein NifD/coenzyme F430 biosynthesis subunit CfbD
	COG1348*	NifH/CfbC	Nitrogenase ATPase subunit NifH/coenzyme F430 biosynthesis subunit CfbC
Photosynthetic carbon reduction cycle	COG0191	Fba	Fructose/tagatose bisphosphate aldolase
(i.e., Calvin cycle)	COG0057	GapA	Glyceraldehyde-3-phosphate dehydrogenase/erythrose-4-phosphate dehydrogenase
	COG1494	GlpX	Fructose-1,6-bisphosphatase/sedoheptulose 1,7-bisphosphatase or related protein
	COG0126	Pgk	3-phosphoglycerate kinase
	COG3954	PrkB	Phosphoribulokinase
	COG1850	RbcL	Ribulose 1,5-bisphosphate carboxylase, large subunit, or a RuBisCO-like protein
	COG4451	RbcS	Ribulose bisphosphate carboxylase small subunit
	COG0036	Rpe	Pentose-5-phosphate-3-epimerase
	COG0120	RpiA	Ribose 5-phosphate isomerase
	COG0698	RpiB	Ribose 5-phosphate isomerase RpiB
	COG0021	TktA	Transketolase
	COG0149	TpiA	Triosephosphate isomerase
Cyanotoxin production	COG3321	PksD	Acyl transferase domain in polyketide synthase (PKS) enzymes
	COG1020	EntF	EntF, seryl-AMP synthase component of non-ribosomal peptide synthetase

*The DNA sequences encoding chlorophyllide reductase subunit were manually removed and only the DNA sequences encoding nitrogenase were used for the analysis.

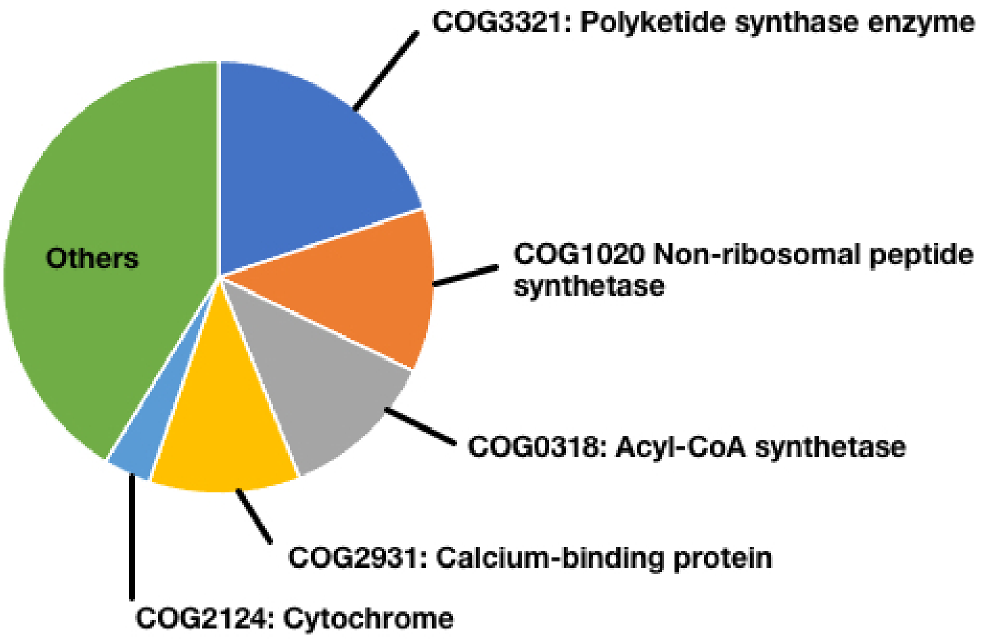

Supplementary Figure S1. Profile of clusters of orthologous groups (COGs) found in the 2014 metagenome data.

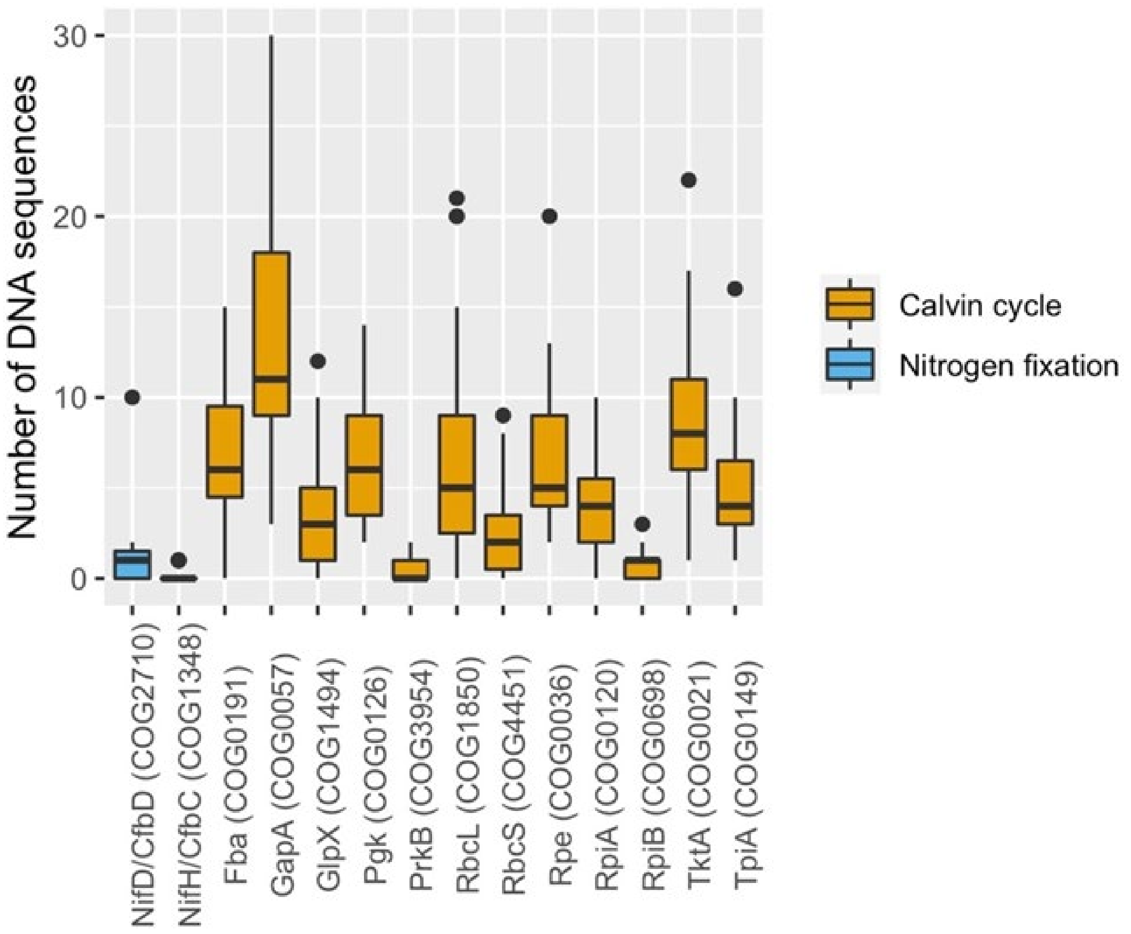

Supplementary Figure S2. The number of DNA sequences associated with nitrogen fixation and the photosynthetic carbon reduction cycle (i.e., Calvin cycle) in all metagenome data.
